# The role of the Pin1-*cis* P-tau axis in the development and treatment of vascular contribution to cognitive impairment and dementia and preeclampsia

**DOI:** 10.3389/fcell.2024.1343962

**Published:** 2024-04-02

**Authors:** Chenxi Qiu, Zhixiong Li, David A. Leigh, Bingbing Duan, Joseph E. Stucky, Nami Kim, George Xie, Kun Ping Lu, Xiao Zhen Zhou

**Affiliations:** ^1^ Department of Medicine, Beth Israel Deaconess Medical Center, Harvard Medical School, Boston, MA, United States; ^2^ Departments of Biochemistry and Oncology, Schulich School of Medicine and Dentistry and Robarts Research Institute, Western University, London, ON, Canada; ^3^ Department of Genetics, Harvard Medical School, Boston, MA, United States; ^4^ Department of Biological Sciences, University of Pittsburgh, Pittsburgh, PA, United States; ^5^ Departments of Pathology and Laboratory Medicine, Schulich School of Medicine and Dentistry, and Lawson Health Research Institute, Western University, London, ON, Canada

**Keywords:** Pin1, tau, tauopathies, cistauosis, vascular dementia, Alzheimer’s disease, stroke, preemclampsia

## Abstract

Tauopathies are neurodegenerative diseases characterized by deposits of abnormal Tau protein in the brain. Conventional tauopathies are often defined by a limited number of Tau epitopes, notably neurofibrillary tangles, but emerging evidence suggests structural heterogeneity among tauopathies. The prolyl isomerase Pin1 isomerizes *cis* P-tau to inhibit the development of oligomers, tangles and neurodegeneration in multiple neurodegenerative diseases such as Alzheimer’s disease, traumatic brain injury, vascular contribution to cognitive impairment and dementia (VCID) and preeclampsia (PE). Thus, *cis* P-tau has emerged as an early etiological driver, blood marker and therapeutic target for multiple neurodegenerative diseases, with clinical trials ongoing. The discovery of *cis* P-tau and other tau pathologies in VCID and PE calls attention for simplistic classification of tauopathy in neurodegenerative diseases. These recent advances have revealed the exciting novel role of the Pin1-*cis* P-tau axis in the development and treatment of vascular contribution to cognitive impairment and dementia and preeclampsia.

## Vascular contribution to cognitive impairment and dementia (VCID)

VCID is featured with neurovascular insults, pathology reminiscent of Alzheimer’s disease (AD) and cognitive decline (Gorelick et al., 2011; Zlokovic, 2011; Iadecola, 2013; OBrien and Thomas, 2015; Snyder et al., 2015). The most common etiology of dementia includes mixed vascular and AD pathologies (Schneider et al., 2004; Schneider et al., 2007; Schneider et al., 2009; James et al., 2012). A critical pathological component of VCID is diffuse white matter lesions, which correlate with cognitive impairment (Pantoni and Garcia, 1997; Medana and Esiri, 2003; Esiri and Nagy, 2007). White matter primarily consists of axonal bundles ensheathed with myelin generated by mature oligodendrocytes, and plays an important role in passing signals between different areas of gray matter. Furthermore, cerebral endothelial cells may support neuronal and oligodendroglial function by secreting trophic factors (Guo et al., 2008; Arai and Lo, 2009). Although there are many potential clinical triggers, brain injury caused by lacunar and larger cerebral infarcts (Snowdon et al., 1997), other hypoperfusion and ischemia (Iadecola, 2004; Garcia-Alloza et al., 2011; Luitse et al., 2012; Rincon et al., 2014) are major vascular factors that contribute to the development of white matter dysfunction, chronic neurodegeneration and dementia (Zlokovic, 2011; Iadecola, 2013; OBrien and Thomas, 2015; Snyder et al., 2015). White matter tracts are especially vulnerable to vascular insults because of their location at the border between different vascular territories (De Reuck, 1971) and their vasculature is highly susceptible to risk factors (Brown and Thore, 2011). However, as compared with neurodegeneration in gray matter, white matter pathophysiology remains relatively understudied, and molecular and cellular mechanisms that connect vascular insults to white matter lesions and delayed neurodegeneration are incompletely understood.

Intercellular interaction among different cell types is critical to maintain white matter function (Itoh et al., 2015). However, under the pathological conditions, this trophic coupling among endothelial cells, oligodendrocytes and neurons may be disrupted, resulting in white matter dysfunction. Iadecola (Iadecola, 2013) has proposed that oxidative stress-induced endothelial dysfunction is likely an early event leading to white matter lesions. Endothelial dysfunction leads to reductions in resting cerebral blood flow (CBF) in the marginally perfused white matter and alterations in the permeability of the blood-brain barrier (BBB). Hypoperfusion and BBB disruption in turn lead to additional oxidative stress by inducing tissue hypoxia and extravasating plasma proteins. Tissue edema resulting from increased BBB permeability may exacerbate these alterations by compressing blood vessels and further reducing CBF, forming a vicious positive feedback cycle. Tissue hypoxia and oxidative stress subsequently activate inflammatory pathways, leading to production of cytokines and adhesion molecules in vascular cells, reactive astrocytes and activated microglia. Hypoxia, oxidative stress and inflammation damage the neurovascular units made of neuronal axons, oligodendrocytes and endothelial cells, and eventually lead to progressive degeneration in axons, myelin and endothelial cells in VCID (Zlokovic, 2011; Iadecola, 2013; OBrien and Thomas, 2015; Snyder et al., 2015).

Neurofibrillary tangles composed of hyperphosphorylated tau is a neuropathological hallmark of AD but not VCID (Mattson, 2004; Goedert and Spillantini, 2006; Roberson and Mucke, 2006; Ballatore et al., 2007; Spires-Jones et al., 2009). Pure VCID human brains were pathologically defined with absence of obvious tau tangle pathology (Gorelick et al., 2011; Zlokovic, 2011; Iadecola, 2013; OBrien and Thomas, 2015; Snyder et al., 2015). Although VCID is not conventionally viewed as a tauopathy, absence of some tau epitopes did not rule out the possibility that other pathogenic tau species could be involved. It has been recently reported that several tau epitopes are increased in VCID patients and mouse models with cerebral hypoperfusion (Castillo-Carranza et al., 2017; Faraco et al., 2019; Qiu et al., 2021; Karakaya et al., 2023; Wang et al., 2023). Importantly, targeting such tau epitopes prevents neuroinflammation, cognitive impairment and other behavioral dysfunctions in mice with cerebral hypoperfusion induced by surgery (Qiu et al., 2021) or high-salt diet (Faraco et al., 2019), revealing important contribution of tau pathologies to the development and progression of VCID.

## Preeclampsia (PE)

PE is a hypertensive disease that occurs frequently in pregnant women and is often associated with cognitive impairment and dementia (Karrar and Hong, 2023). PE has an incidence rate of up to 8% in pregnancy complications, resulting in over 550,000 maternal and fetal deaths worldwide (Karrar and Hong, 2023). The pathogenesis of PE can be divided into two stages: placental abnormalities and maternal syndrome. Placental dysfunction is mainly influenced by genetic, maternal, and immune factors, leading to the generation of anti angiogenic factors such as soluble fms like tyrosine kinase 1 (sFlt-1), soluble endoglin (sEng), and other inflammatory mediators, thereby promoting the progression of PE. PE usually manifests as newly developed hypertension and proteinuria in late pregnancy, which can progress to multiple organ dysfunction, including chronic diseases such as liver, kidney, and brain (Phipps et al., 2019; Rana et al., 2019). Among the most serious prognostic diseases are the mild cognitive impairment and dementia in mothers and their offsprings (Tuovinen et al., 2012; Basit et al., 2018; Lu et al., 2019; Andolf et al., 2020). Tau is one of the candidate biomarkers for diagnosing and predicting PE complications (Bergman et al., 2022; Friis et al., 2022). It has been shown that phosphorylated tau is superior to total tau in predicting the cognitive ability of PE patients (Wang et al., 2023). However, these evidences are largely observational and do not reveal the molecular mechanisms and therapeutic potential of phosphorylated Tau. Recently, we revealed that cis conformation of phosphorylated Thr231 of Tau (*cis* P-tau) is a central circulating etiological driver in PE, and the cis P-tau specific monoclonal antibody (cis mAb) has potential for early PE diagnosis and treatment (Jash et al., 2023), suggesting that PE may be another non-conventional tauopathy. Below we review studies relevant to the discovery of *cis* P-tau, one of the major deleterious tau species that arises early and disrupts the function of the neurovascular unit to connect initial vascular insults to the development of white matter lesions, delayed neurodegeneration and neurologic defects in tauopathies such as VCID and PE.

## Pin1 isomerizes P-tau to inhibit the development of tau pathology and neurodegeneration

**FIGURE 1:**
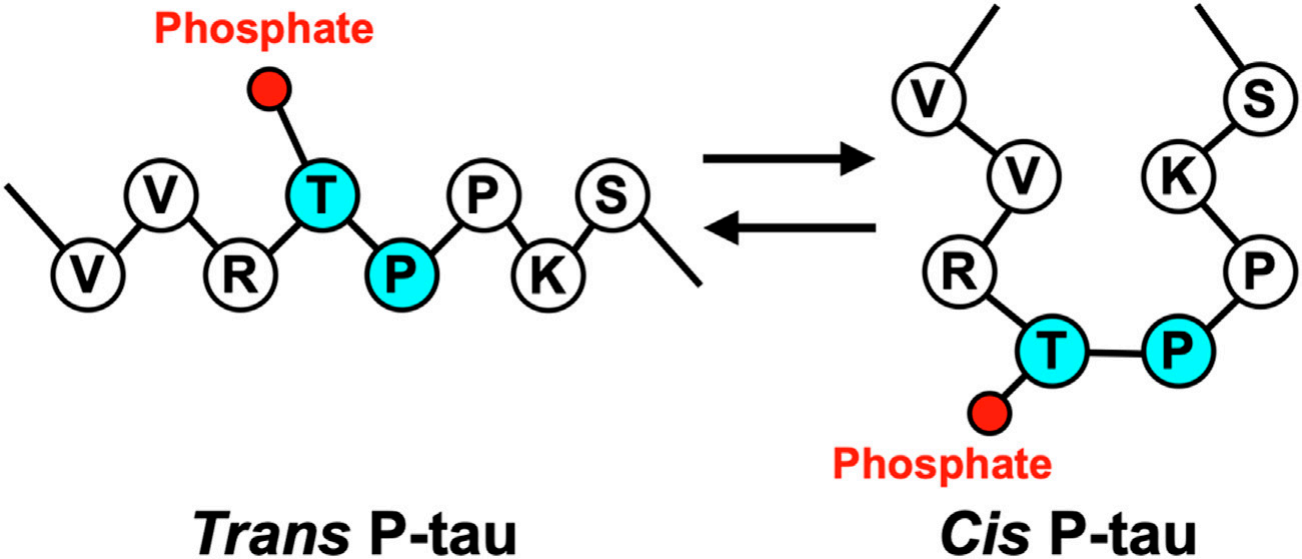
Tau proline 232 isomerizes between the trans and cis conformations.

*Cis* P-tau is featured with its Pro232 in a rare *cis* conformation, which likely affects the Thr231 phosphorylation and Tau function (Figure 1). Proline-directed Ser/Thr phosphorylation is a key signaling mechanism in cells (Blume-Jensen and Hunter, 2001; Pawson and Scott, 2005). Many pSer/Thr-Pro motifs exist in *cis* or *trans* conformations, and their conversion and function are regulated by the unique prolyl isomerase Pin1 (Lu et al., 1996; Ranganathan et al., 1997; Yaffe et al., 1997). Pin1 is tightly regulated (Wulf et al., 2001; Lu et al., 2002; Ryo et al., 2002; You et al., 2002; Hamdane et al., 2006; Lee et al., 2011) and its deregulation can contribute to various diseases, notably including AD, TBI, VCID, and PE (Nakamura et al., 2012; Kondo et al., 2015; Albayram et al., 2017; Albayram et al., 2019; Qiu et al., 2021; Jash et al., 2023). Pin1 acts on the pThr231-Pro motif (P-tau) in tau (Lu et al., 1999) to facilitate P-tau dephosphorylation by Protein phosphatase 2A (Lu et al., 1999), a *trans*-proline directed phosphatase (Zhou et al., 2000; Liou et al., 2003). Pin1 facilitated P-tau dephosphorylation leads to increased tau degradation; accordingly, various hyperphosphorylated tau, including P-tau, are resistant to degradation. Furthermore, Pin1 restores the physiological function of tau to promote microtubule assembly (Lu et al., 1999), a function of tau that is inhibited by hyperphosphorylation. In contrast, Pin1 has no effect on tau T231A mutant (Lu et al., 1999; Zhou et al., 2000; Liou et al., 2003; Lim et al., 2008), although Pin1 can bind and isomerize other motifs *in vitro* (Smet et al., 2004; Kimura et al., 2013; Eichner et al., 2016), suggesting that the primary Pin1 target site in Tau *in vivo* is pT231-Pro.

Genetic and pathological data have supported a critical role of Pin1 in neuroprotection. Pin1 −/− mice display age-dependent tau-related pathologies and neurodegeneration (Liou et al., 2003; Pastorino et al., 2006; Cancino et al., 2013), while moderate Pin1 overexpression prevents wild type tau pathology in mice (Lim et al., 2008). In humans, Pin1 can be inhibited by various mechanisms including downregulation/sequestration (Lu et al., 1999; Thorpe et al., 2001; Thorpe et al., 2004; Hamdane et al., 2006) Ser71 phosphorylation (Lee et al., 2011; Kim et al., 2014) and Cys113 oxidation (Sultana et al., 2006; Chen et al., 2015). For example, oxidative stress from the AD brain could oxidize Pin1 Cys113 and inactivate Pin1, leading to the loss of regulation on Tau and APP and increased neurofibrillary pathology (Lee et al., 2011; Kim et al., 2014). Furthermore, cerebral ischemic insults activate DAPK1, which phosphorylates Pin1 Ser71 and leads to Pin1 inhibition, cis P-tau induction, pathology, neuroinflammation and memory impairment (Sultana et al., 2006; Chen et al., 2015; Qiu et al., 2021). It is worth noting that the human PIN1 gene is located at 19p13.2, a new late onset AD locus distinct from ApoE4 (Wijsman et al., 2004). PIN1 SNPs that reduce Pin1 expression (Lu et al., 2009) are associated with increased risk for AD in an Italian cohort (Segat et al., 2007), although not in others (Nowotny et al., 2007; Ma et al., 2012) whereas a different SNP that prevents Pin1 suppression by the brain-selected AP4 is associated with delayed onset of AD (Ma et al., 2012). In addition, pT231-tau (P-tau), the Pin1 substrate, is induced at early stages of AD prior to tangle formation (Jicha et al., 1997; Augustinack et al., 2002; Luna-Munoz et al., 2005; Luna-Munoz et al., 2007). CSF P-tau is an early biomarker that correlates with cognitive decline (Buerger et al., 2002; Buerger et al., 2005), neocortical tangle accumulation (Buerger et al., 2006), hippocampal atrophy rate (Hampel et al., 2005), and predicts progression from mild cognitive impairment (MCI) to AD (Ewers et al., 2007; Blennow et al., 2010; Hampel et al., 2010). Thus, pT231-tau (P-tau) is likely an early disease driver in AD.

## Distinct functions of cis P-tau and trans P-tau are revealed by conformation-specific antibodies

Pin1 facilitates the interconversion of *cis* and *trans* pT231-Pro motif in Tau, but it was unclear from the genetic and functional data whether *cis* P-tau or *trans* P-tau was pathogenic. To distinguish the functions of *cis* P-tau and *trans* P-tau, we generated *cis* and *trans* P-tau isomer-specific antibodies using an innovative approach leveraging the distinct structural properties of prolines (Nakamura et al., 2012; Kondo et al., 2015). We started by substituting proline232 with homoproline (Pip), which has a six-membered ring and leads to an increased proportion of cis peptide bond conformation (∼74%) (Table 1) (Nakamura et al., 2012). Using this peptide, we successfully produced antibodies that recognized P-tau with either trans or cis comformation. For further conformational specificity, we synthesized cis-locked or trans-locked peptides for selection and counter-selection of cis or trans P-tau specific antibodies. Peptides were locked in cis using pThr231-Dmp (5,5-dimethylproline) (∼96% in cis conformation) and locked in trans by pThr231-Ala (0% in cis), as validated by NMR (Table 1) (Nakamura et al., 2012). After selection and purification, the specificity of these cis- and trans-specific antibodies was confirmed by ELISA, demonstrating negligible cross-reactivity and high affinity for their respective cis and trans pT231-tau peptides. These antibodies did not react with an unphosphorylated Thr231-Pro tau peptide but showed strong reactivity to a wild-type pThr231-Pro tau peptide, highlighting their high phosphorylation and conformational specificity (Nakamura et al., 2012; Kondo et al., 2015).

**TABLE 1:** Peptides used to generate and purify cis P-tau and trans P-tau specific antibodies.

Peptide name	Peptide sequence	Percentage of cis conformation
Wild-type phosphorylated Thr231-Pro tau	KVAVVRpTPPKSPS	9
cis lock-in phosphorylated Thr231-Dmp tau	KVAVVRpT (5,5-dimethyl-L-proline) PKSPS	96
trans lock-in phosphorylated Thr231 tau (tau P232A)	KVAVVRpTAPKSPS	0
cis preferred phosphorylated Thr231-Homoproline (pThr231-Pip)	CKKVAVVRpT (Pip)PKSPSSAK	74

**FIGURE 2:**
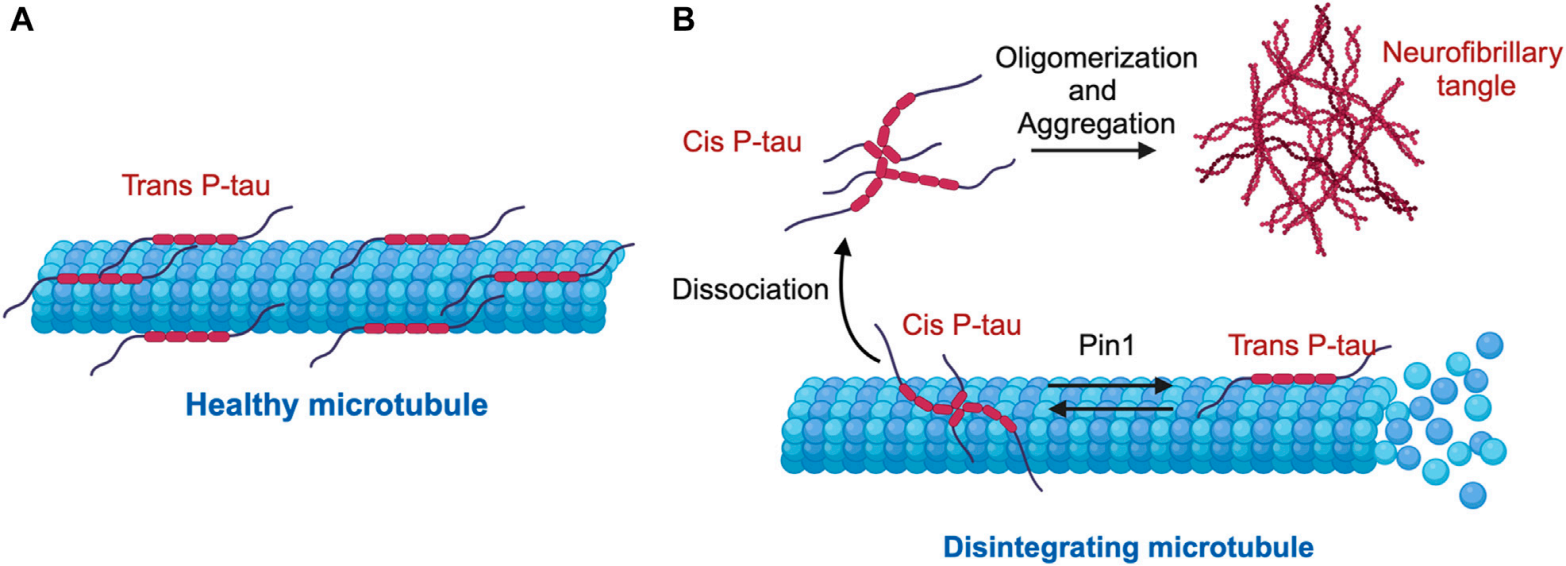
Mechanism of cis-trans isomerization of pT231-Tau (P-tau) in the regulation of microtubule stability and function. **(A)** Under physiological conditions, P-tau is predominantly in the physiological trans P-tau form, which binds to and stabilizes microtubules. **(B)** Under stress conditions, cis P-tau is induced, while the prolyl isomerase Pin1 binds and catalyzes the isomerization of the pathogenic cis P-tau to the physiological trans P-tau. In several neurodegenerative diseases such as TBI, VaD, PE, and AD, loss or inhibition of Pin1 leads to excessive accumulation of cis P-tau, which dissociates from microtubules, oligomerizes and eventually aggregates into neurofibrillary tangles, leading to destabilized microtubules.

Using these antibodies, we dissected the functions of *cis* P-tau and *trans* P-tau (Nakamura et al., 2012; Kondo et al., 2015). *Cis,* but not *trans,* P-tau loses its classical microtubule assembly function, confering resistance to dephosphorylation and degradation and also promoting its tendency to aggregation (Figure 2) (Nakamura et al., 2012). *Cis* to *trans* conversion does not occur spontaneously, but is significantly accelerated by Pin1 (Figure 2) (Nakamura et al., 2012). Thus, *cis,* but not *trans,* P-tau is likely the pathogenic form of P-tau, and antibodies targeting *cis* P-tau could have potential applications in both the diagnosis and treatment of various neurodegenerative diseases associated with P-tau (Nakamura et al., 2012). Below we summarize recent work highlighting the role of *cis* P-tau as an early, pathogenic conformer in AD, TBI, VCID, and PE, as well as the therapeutic potential of the monoclonal antibody targeting *cis* P-tau (*cis* mAb).

## *Cis* P-tau is an early pathogenic tau conformation and blood biomarker in AD, TBI, VCID, and PE

*Cis* P-tau has emerged as an early pathogenic tau conformation in multiple neurodegenerative diseases since its initial discovery. First, we hypothesized that cis P-tau is pathogenic in AD, because pT231-Tau (P-tau) is induced at early stages of AD and Pin1 −/− mice conferred age-dependent tauopathy and neurodegeneration (Liou et al., 2003; Pastorino et al., 2006; Cancino et al., 2013). Indeed, in hTau mice, where endogenous mouse Tau is replaced with human Tau (Duff et al., 2000; Andorfer et al., 2003), *cis* P-tau is induced at 3-months of age, prior to formation of early tangles (Qiu et al., 2021). In patients, *cis* P-tau is induced early in MCI neurons and is specifically induced at the dystrophic neurites of degenerating neurons (Nakamura et al., 2012), correlating well with cognitive deficits as AD progresses (Davies et al., 1987; DeKosky and Scheff, 1990; Scheff et al., 1990; Terry et al., 1991; Masliah et al., 1992; Coleman and Yao, 2003; Thies and Mandelkow, 2007). Moreover, plasma *cis* P-tau is induced extremely early in AD patients (Ashton et al., 2021; Milà-Alomà et al., 2022; Shiravandi et al., 2022), being the first blood biomarker able to distinguish between Braak stage I/II in incipient AD from health controls (Ashton et al., 2021) and to reach the abnormal level with the lowest Aβ burden in pre-clinical AD patients (Milà-Alomà et al., 2022).

TBI is another classical tauopathy when the timing of disease onset can be precisely modeled in mice (Kondo et al., 2015). *cis* P-tau is induced as early as 12 h after single severe closed head TBI in the mouse model (Kondo et al., 2015). After closed head TBI in mice and stress *in vitro*, neurons acutely produce *cis* P-tau notably at axons, which disrupts axonal microtubules and axonal transport, spreads to other neurons, and leads to apoptosis. In patients, cis P-tau can be induced as early as 8 h after motor vehicle injury (Albayram et al., 2017). Furthermore, we find robust *cis* P-tau in human chronic traumatic encephalopathy (CTE) brains (Kondo et al., 2015). Tangles are a neuropathological signature of CTE following sport and military TBI (Omalu et al., 2005; Blennow et al., 2012; Goldstein et al., 2012; DeKosky et al., 2013; McKee et al., 2013; Smith et al., 2013), but tau tangle pathology is not readily detectable acutely after closed head TBI in humans and mouse models (Smith et al., 2003; Yoshiyama et al., 2005; Flierl et al., 2009; Tran et al., 2011; Goldstein et al., 2012; Kane et al., 2012; Mannix et al., 2013; Ojo et al., 2013), although tau oligomers can be detected after open head severe TBI (Hawkins et al., 2013). Finally, *cis* P-tau in the CSF (Albayram et al., 2017) or P-tau in the plasma (Rubenstein et al., 2017) also correlate with TBI injury severity and clinical outcome in acute and chronic phases, with a 10- to 15-fold increase in plasma P-tau within 24 h after severe TBI (Rubenstein et al., 2017).

Discovery of cis P-tau at an early stage of AD and TBI, long before the presence of neurofibrillary tangles, suggested that absence of classical tangles does not exclude a neurodegenerative disease as a tauopathy. We next set out to ask if cis P-tau is implicated in VCID, because VCID pathology often co-occurs with AD in the late stage. We hypothesize that cis P-tau may be similarly induced at an early stage of VCID, prior to tangle pathology. Notably, *cis* P-tau is significantly induced in various cohorts of pure VCID patients prior to co-emergence of AD pathologies (Qiu et al., 2021). Furthermore, we set out to ask if cis P-tau is induced early in mice after bilateral common carotid artery stenosis (BCAS), an experimental approach widely used to induce chronic cerebral hypoperfusion and model VCID by partial occlusion of both common carotid arteries in mice (Shibata et al., 2004). The reduced blood flow to the brain leads to the vascular pathology and subsequently cognitive impairment observed in VCID, allowing for the study of its mechanisms and potential therapeutic interventions (Shibata et al., 2004; Shibata et al., 2007). We observed robust *cis* P-tau induction at 2 weeks after the BCAS sugery, an early time point prior to significant demyelination and cognitive decline (Qiu et al., 2021).

Finally, implication of cis P-tau in VCID, a non-conventional tauopathy, suggested that there might be a broader spectrum of neurodegenerative diseases driven by cis P-tau and other toxic Tau species. Indeed, *cis* P-tau and several other phosphorylated tau epitopes are significantly induced in PE placental tissue and serum as compared to that of normal pregnant women (Jash et al., 2023). Both *in vivo* and *in vitro* studies have shown that *cis* P-tau, sFlt-1, and sEng are induced in the primary human trophoblast in response to hypoxia and serum, as well as trophoblast invasion and interruption of intravascular activity in PE patients (Jash et al., 2023), suggesting that blood cis P-tau can be also an early biomarker for early and late-onset PE (Jash et al., 2023). Therefore, *cis* P-tau is an early tau conformation prior to oligomers and tangles in AD, TBI, VCID, and PE.

## *Cis* P-tau monoclonal antibody ameliorates the progression of TBI, AD, VCID and PE

To explore the therapeutic potential of targeting *cis* and *trans* P-tau, we generated, screened and purified *cis* and *trans* P-tau monoclonal antibodies (*cis* mAb or *trans* mAb) with high affinity (Cis mAb: 0.27 nM, trans mAb: 42.1 nM) (Kondo et al., 2015), and evaluated their therapeutic potential in a range of TBI, AD-like, and VCID mouse models (Kondo et al., 2015; Albayram et al., 2017; Albayram et al., 2019; Qiu et al., 2021). We treated single-severe closed head TBI mice (54 g weight drop from 60 inches (Albayram et al., 2017)) with 200 µg cis mAb at 4 h after TBI, followed by weekly treatment until 2-month post-TBI and bi-weekly treatment (every other week) until 4-month TBI (Albayram et al., 2017). In parallel, we treated repetitive moderate closed head TBI mice (54 g weight drop from 34 inches per impact, and 7 impact over 9 days) with one dose after each impact, followed by weekly treatment until 2-month post-TBI and bi-weekly treatment (every other week) until 4-month TBI. In both TBI models, *cis* mAb blocks early cistauosis, prevents subsequent tau pathology, and restores TBI-related structural and functional outcomes (Kondo et al., 2015; Albayram et al., 2017; Albayram et al., 2019). Specifically, *cis* mAb reversed axonal and mitochondrial ultrastructural pathologies, cortical and hippocampal long-term potentiation impairment, risk-taking behavior, voiding dysfunction and working memory defects in TBI mouse models (Kondo et al., 2015; Albayram et al., 2017; Albayram et al., 2019). The *cis* mAb efficacy is consistent with the earlier evidence that Tau knockout prevents memory defects and axonopathy after repetitive TBI in mice (Cheng et al., 2014).

Second, in AD-like tauopathy hTau mice, which develops age-related tau pathologies and neurodegeneration, *cis* mAb (300 µg per mouse, intraperitoneally (i.p.) for the first 4 months, followed by every other week treatment) prevents formation of *cis* P-tau, tangles, neurodegeneration, demyelination and impairment in working memory and spatial learning/memory (Qiu et al., 2021). Furthermore, extended *cis* mAb treatment of 13-month-old hTau mice (300 µg per mouse, intraperitoneally (i.p.) weekly), which already developed tau pathologies and memory deficits, is sufficient to specifically eliminate *cis* P-tau, prevent further neuronal loss and rescue working memory impairment (Qiu et al., 2021). It is worth noting that *cis* mAb treatment does not reduce neurofibrillary tangles (Qiu et al., 2021), consistent with the hypothesis that soluble Tau, but not the insoluble Tau fibrils, is neurotoxic (Goedert et al., 2017; Hyman, 2023).

Third, *cis* mAb confers notable neuroprotection to the VCID mouse model (Qiu et al., 2021). *Cis* mAb treatment (300 µg per mouse, intraperitoneally (i.p.), every 3 days for four times, and then 150 µg per mouse every week afterwards) specifically eliminates *cis* P-tau (without perturbing total tau) in the BCAS mice, reducing neuroinflammation, demyelination and rescuing impaired hippocampal long-term potentiation (Qiu et al., 2021). Furthermore, *cis* mAb rescued the working memory impairment and chronically induced risk-taking behavior in BCAS mice in multiple behavioral assays (Qiu et al., 2021). In addition, BCAS induced diverse cortical cell type-specific transcriptomic changes, many of which resembled changes seen in AD patients, featuring alteration of pathways in myelination, axon/synapses function, microtubule related function and GTP signaling (Qiu et al., 2021). Strikingly, 85%–90% of the global alterations are recovered by *cis* mAb, and the extent of recovery in different cell types is correlated with the cell type-specific tau expression. This is consistent with the hypothesis that *cis* P-tau confers toxicity through prion-like propagation and requires endogenous Tau, thus the extent of *cis* P-tau toxicity correlates with endogenous Tau expression levels. Therefore, *cis* mAb is highly effective in eliminating *cis* P-tau, recovering pathology, behavior and cell type-specific transcriptome of BCAS mice.

Finally, depletion of *cis* P-tau in PE serum significantly inhibits the ability of PE serum to induce all PE-like pathological and clinical features in humanized tau mice during pregnancy (Jash et al., 2023). Serum from PE patients, but not healthy pregnant individuals, when injected (i.p.) into the pregnant hTau mice, leads to PE symptoms, including accumulation of protein aggregates in the junctional zone, elevated blood pressure, proteinuria, fetal growth restriction, and glomerular endotheliosis (Aoki et al., 2018). Notably, when the serum from PE patients were depleted for *cis* P-Tau by cis mAb, the PE related pathology and symptoms were largely rescued, and normal pregnancy was restored (Jash et al., 2023).Taken together, *cis* P-tau is an early driver of neurodegeneration and a potential therapeutic target in TBI, AD, VCID and PE. It is worth noting that *cis* mAb is the only Alzheimer’s clinical drug candidate that has potential for brain injury, VCID, and preeclampsia, which are among the best-known risk factors for dementia.

## Purified soluble *cis* P-tau is a proteotoxic driver of neurodegeneration

Cortical injection of brain lysates or tau aggregates leads to progressive neurodegeneration in young WT mice (Clavaguera et al., 2009; Clavaguera et al., 2013; Clavaguera et al., 2014; Sanders et al., 2014; Kaufman et al., 2016). We next ask if purified *cis* P-tau is sufficient to induce neurodegeneration in neuronal culture and in wild-type animals. We purified *cis* P-tau from TBI mouse brains using *cis* mAb, and found that purified *cis* P-tau induced much higher neurotoxicity than recombinant Tau in neuronal culture. Furthermore, the *cis* P-tau induced neurotoxicity is blocked by *cis* mAb treatment or pan-caspase inhibitor, suggesting that *cis* P-tau induced neurotoxicity is caspase dependent and may be linked to neuronal apoptosis. Thus, purified *cis* P-tau, but not recombinant tau, induced neuronal apoptosis and could be blocked by *cis* mAb.

To test if purified *cis* P-tau is sufficient to induce progressive neurodegeneration and brain dysfunction in wild-type animals, we stereotaxically injected purified *cis* P-tau (or recombinant tau as a control) bilaterally into the cortex of 3-month-old WT mice, and evaluated the pathological and behavioral outcomes at 1 or 10 months after the injection. We observed progressive prion-like tau spreading, neuroinflammation and behavioral dysfunction. At 1 month post-injection, *cis* P-tau, but not recombinant tau control, induces apparent risk-taking behaviors reminiscent of TBI mice, as assayed by elevated plus maze and bright-light open field assays. At 10 months post-injection, *cis* P-tau induces a range of somatosensory motor deficits and working memory impairment in addition to the persistent apparent risk-taking behavior. Consistent with the behavioral alteration, *cis* P-tau appears to propagate across cortical regions and induce subsequent tau pathologies (*e.g.*, tangles) and ultra-structural pathologies. *Cis* mAb treatment eliminated *cis* P-tau and prevented various pathological and behavioral changes. Finally, single-nucleus RNA-seq revealed that injection of purified *cis* P-tau induces a subset of cell type-specific transcriptomic changes in BCAS mice, recapitulating altered genes in myelination, axon/synapse function and microtubule related function. Strikingly, the *cis* P-tau induced transcriptomic changes in the excitatory neurons have significant overlap with recently reported excitatory neuronal changes in patients with early, but not late AD pathologies. The vast majority of these changes can be rescued by *cis* mAb, and injection of purified *cis* P-tau into Tau knock-out mice does not induce pathology and behavioral alternations, consistent with requirement of endogenous Tau for the prion-like propagation of cis P-tau.

## Tauopathies and beyond

Tauopathies are a series of neurodegenerative diseases characterized with neurofibrillary tangles or well-known Tau epitopes. Recent advances in cryo-EM structures of Tau fibrils enabled structurally based classification of Tauopathies, revealing distinct folds for different tauopathies (Fitzpatrick et al., 2017; Falcon et al., 2018a; Falcon et al., 2018b; Falcon et al., 2019; Zhang et al., 2020; Shi et al., 2021). However, it has been increasingly recognized that the toxic Tau species may be soluble but not aggregated (Goedert et al., 2017; Hyman, 2023). It is thus critical to evaluate which Tau epitopes may underlie the disease progression and to revisit how Tauopathies are defined. VCID and PE are two such examples. VCID was not conventionally considered as a tauopathy, largely due to the absence of neurofibrillary tangles in pure VCID patients with cognitive impairment. While most tauopathies are age-dependent and related to brain dysfunction, PE represents the first example where *cis* P-tau drives disease development outside of the brain and in a younger population represented by pregnant women. We and Faraco et al. have shown that absence of tangles does not preclude the pathogenic roles of other Tau PTMs such as pSer202, pThr205, P-tau (pThr231) and *cis* P-tau (Faraco et al., 2019; Qiu et al., 2021). Tau can be heavily modified by post-translational modifications, with >80 potential PTM sites, far beyond the known epitopes (Goedert, 2005; Goedert et al., 2017). The potential pathogenic role of cis P-tau in AD, TBI and non-conventional tauopathies (VCID and PE) reviewed here may suggest a broader spectrum of neurodegenerative diseases that could be driven by pathogenic Tau.
